# Efficacy of Tenoxicam, Paracetamol, and Their Combination in Postoperative Pain After Double-Jaw Surgery

**DOI:** 10.7759/cureus.44195

**Published:** 2023-08-27

**Authors:** Seher Orbay Yaşli, Dilek Günay Canpolat, Fatma Doğruel, Ahmet E Demirbaş

**Affiliations:** 1 Anesthesiology in Oral and Maxillofacial Surgery, Erciyes University Faculty of Dentistry, Kayseri, TUR; 2 Oral and Maxillofacial Surgery, Erciyes University Faculty of Dentistry, Kayseri, TUR

**Keywords:** opioid, analgesics, tenoxicam, postoperative pain, pain management, orthognathic surgical procedures

## Abstract

Introduction

Orthognathic surgical procedures include a series of surgical operations in which interventions are applied to the maxilla, mandible, or both for occlusal or aesthetic concerns due to facial skeletal development deformities. Double-jaw surgeries have the highest pain scores, in which both maxilla and mandible bones are intervened. This study aimed to compare the efficacy of individual applications of paracetamol and tenoxicam with their combined application on postoperative pain and opioid consumption in patients undergoing double-jaw surgery.

Methods

In this randomized, double-blind study, 60 patients undergoing double-jaw surgery were allocated into three groups, with each having 20 patients: the paracetamol group, the tenoxicam group, and the paracetamol-tenoxicam combination group. Pain intensity was evaluated using the visual analogue scale (VAS) at intervals of 30 minutes, 60 minutes, 120 minutes, and again at the 24th postoperative hour. Additionally, the consumption of opioids and other rescue analgesics was documented over the 24-hour postoperative period.

Results

The VAS values at 30 minutes, 60 minutes, and 24 hours were lower in the paracetamol-tenoxicam group compared to the other groups (p<0.001). The need for a rescue analgesic drug in the first 24 hours was not observed in the tenoxicam and paracetamol-tenoxicam groups.

Conclusion

It was concluded that both tenoxicam and paracetamol-tenoxicam combinations, especially the tenoxicam-paracetamol combination, were good options for postoperative analgesia in patients with double-jaw surgery.

## Introduction

Postoperative pain generally occurs due to inflammation and edema related to tissue trauma. Many factors, such as cautery-related burns, surgical incisions, dissections, and instrumental procedures such as cutting, stretching, or compression, cause surgical tissue trauma. The transmission pathways of pain begin with specific receptors that release local mediators in the periphery. Subsequently, the pain signals are transmitted to central nervous system structures, particularly the thalamus and cerebral cortex, where they are processed and ultimately perceived as pain. This process, which starts with the reception and transmission of the pain sensation from the periphery and ends with the perception in the upper centers of the nervous system, is quite complex and is called nociception [1]. The nature of nociception and the complex mechanisms that cause surgical pain are also responsible for the inadequacy of unimodal analgesia in postoperative pain. So, the recently adopted approach to postoperative pain management is multimodal analgesia, which is performed using multiple drugs and methods that act through different mechanisms [2]. Tenoxicam is an analgesic, anti-inflammatory, and antipyretic drug with a long duration of action, and is included in the oxicam subgroup of non-steroidal anti-inflammatory drugs (NSAIDs). Tenoxicam has been studied and found effective for many rheumatic diseases, such as rheumatoid arthritis, ankylosing spondylitis, gout, extra-articular disorders, bursitis, tendonitis, and osteoarthritis [3]. In addition, tenoxicam is known to reduce postoperative pain and opioid consumption significantly [4,5]. But to our knowledge, it has yet to be studied in orthognathic surgery. In multimodal analgesia protocols, NSAIDs and paracetamol (acetaminophen) are commonly preferred analgesic drugs. This is because the beneficial role of these medications in pain management has been well documented [6,7].

Orthognathic surgical procedures include a series of surgical operations in which interventions are applied to the maxilla, mandible, or both for occlusal or aesthetic concerns due to facial skeletal development deformities. Double-jaw surgeries have the highest pain scores in which both maxilla and mandible bones are intervened [8]. Compared with other NSAIDs, tenoxicam significantly inhibits proteoglycanase and collagenase activity and positively impacts the overall metabolism of proteoglycans and hyaluronan in cartilage [3,9]. These features led us to hypothesize that tenoxicam may be a useful analgesic drug for orthognathic surgery.

Thus, our hypothesis was that the combined use of paracetamol and tenoxicam could effectively manage postoperative pain, potentially reducing the need for postoperative opioid analgesics. Considering this, the primary aim of this study was to compare the effects of tenoxicam, paracetamol, and tenoxicam-paracetamol combination analgesic applications on the postoperative visual analogue scale (VAS) score of double-jaw surgery patients. In line with this aim, the primary outcome targeted was the postoperative VAS scores. The secondary aim was to investigate the effects of these interventions on the number of both opioid and rescue analgesic drug consumption postoperatively. Correspondingly, the secondary outcomes encompassed the postoperative consumption of both opioid and rescue analgesics.

## Materials and methods

The study was conducted by the Declaration of Helsinki and was carried out with the approval of the Ethics Committee of Erciyes University Faculty of Medicine (2018/303). Written informed consent was taken from all the patients participating in the study. Sixty patients between 18 and 50 years of age, both genders, who were scheduled for double-jaw surgery under general anesthesia and classified as American Society of Anesthesiologists (ASA) risk class I or II were included in the study. Exclusion criteria for the study patients were as follows: having liver or renal dysfunction and known coagulopathy disorder, having psychiatric or medical conditions that might impair communication or compliance with the study procedures, having allergy or contra-indications to the study drugs, and pregnancy. In addition, patients who were planned to undergo additional simultaneous surgical procedures such as genioplasty were not included in the study. The same surgery team performed all surgeries. This study was retrospectively registered on ClinicalTrials.gov (NCT05508451).

Study design, detailed methodology, and follow-up

This is a single-centered, randomized, double-blind study conducted between May 2019 and April 2021. Randomization was carried out using the coin-toss method. Prior to the study, the nurse was briefed about the study design, and she was responsible for the randomization process and setting up the groups, ensuring an unbiased allocation of participants into the three study groups. Treatments consisted of IV infusions of paracetamol 1 g, tenoxicam 20 mg, and a combination of paracetamol and tenoxicam. The nurse, who was privy to the contents of the solutions, including the drugs they contained, recorded this information. Other clinical personnel were not informed about the specific group allocations to maintain the integrity of the study. These solutions were then administered to the patients. The specific drugs contained in each solution were only revealed after the study was completed, at which point the groups were formed accordingly.

Standard monitoring, including pulse oximetry, electrocardiography, and non-invasive blood pressure assessment, was performed for all patients. Anesthesia induction was achieved using fentanyl (1 microgram/kg), propofol (2 mg/kg), and rocuronium (0.5 mg/kg). Additionally, 2% sevoflurane was used for maintenance.

Approximately 30 minutes before the conclusion of the operation, as the mucosal closure began, intravenous solutions containing the designated analgesic drugs (either paracetamol, tenoxicam, or a combination of both) were administered to the patients. These analgesics were infused continuously until the final suture was placed. These solutions were prepared by a nurse to ensure the appropriate concentration and volume for each patient. Vital signs such as heart rate, mean arterial blood pressure, and SpO_2_ were recorded at 5-minute intervals during the application of these solutions. Furthermore, an evaluation of patients' pain severity was conducted upon emergence from anesthesia, following extubation in the operating room. This was assessed using a 5-point verbal scale, with the points defined as follows: (0) none, (1) a little, (2) moderate, (3) a lot, and (4) complete. A modified Aldrete Scoring System was used in the postoperative period to assess recovery. The time to reach the modified Aldrete recovery score of 9 during recovery from anesthesia was noted for each patient.

**Figure 1:**
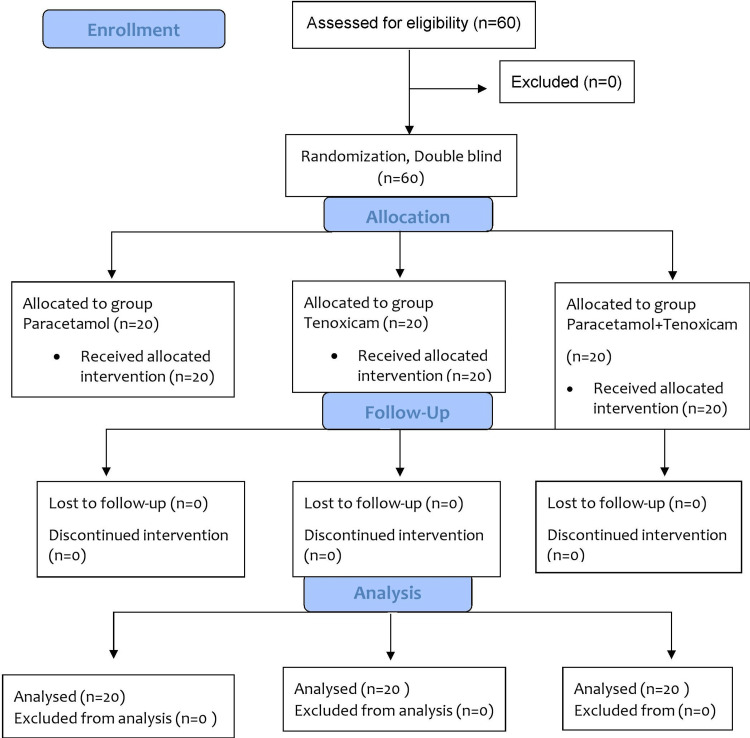
CONSORT flow diagram of the study CONSORT, Consolidated Standards of Reporting Trials

Pain intensity was assessed on a 100-mm VAS, where 0 = no pain and 100 = worst possible pain at 30 minutes and at the first, second, and 24th hour after surgery. The decision to include the 30th-minute evaluation was made based on our study's design and the timing of the drug administration, ensuring an accurate assessment of the initial analgesic effects. In our study, as the primary postoperative analgesic, an opioid analgesic drug, specifically 50 mg IV tramadol, was administered to the patients whenever their VAS scores reached or exceeded 40. This was to ensure immediate pain relief. However, if a patient explicitly requested additional pain relief or if the primary analgesic was not sufficient, we provided rescue analgesia. In such cases, IV paracetamol was chosen as the rescue analgesic and was administered only after a thorough pain assessment to ensure the patient's safety and comfort. The number of both opioid and rescue analgesic drugs administered based on VAS scores was recorded. Postoperative analgesic consumption was specifically monitored and recorded for the initial 24 hours following the surgery to assess the immediate pain management efficacy of the treatments. The amount of maxillo-mandibular movement for each patient was also recorded. Postoperative complications such as nausea, vomiting, arrhythmia, agitation, and surgical duration were the recorded other parameters for the study. The consort flow diagram of the current randomized study is given in Figure 1.

Statistical analyses

Data analysis was performed using the Statistical Package for the Social Sciences (SPSS) software Version 26 (IBM Corp., Armonk, New York, USA). Descriptive statistics were given several units (n), percent (%), mean ± standard deviation, median (M), minimum (min), maximum (max), and interquartile range (IQR). The normal distribution of the data of numerical variables was evaluated using the Shapiro-Wilk normality test. Pearson’s chi-square and Fisher’s exact tests were used to compare groups with categorical variables. If the results of the chi-square analysis were significant, subgroup analyses were performed using a Bonferroni-corrected two-ratio z-test. Comparison of the normally distributed variables between the groups was done using a one-way analysis of variance. Variables that did not show normal distribution were compared using the Kruskal-Wallis analysis. If the Kruskal-Wallis analysis result was significant, the Dunn-Bonferroni test was used as a multiple comparison test. A two-way analysis of variance was used in repeated measurements from general linear models to compare the groups' heart rate and blood pressure values according to the measurement times. In the study, Spearman's rho correlation coefficient was used to examine the relationship between the covariates and the study's primary outcomes, which were the VAS scores and the number of postoperative opioid consumptions. A p-value of <0.05 was considered statistically significant in all comparisons.

A post hoc power analysis was conducted in accordance with the effect sizes proposed by Cohen, using G*Power [10]. With 95% confidence (1-α) and an effect size of 0.86, the power of the test was determined to be 99%. This implies that the data obtained from the sample are reliable and generalizable.

## Results

Age, gender, BMI, and ASA distributions were statistically similar between the groups (p>0.05, Table 1).

**Table 1:** Comparison of descriptive characteristics of groups *Pearson chi-square test. **Kruskal-Wallis analysis test. ***One-way analysis of variance BMI, body mass index; ASA, American Society of Anesthesiologists

Descriptive characteristics	Paracetamol, n=20	Tenoxicam, n=20	Paracetamol + tenoxicam, n=20	p-Value
Gender, n (%)				0.959*
Female	12 (60.0)	11 (55.0)	10 (50.0)	
Male	8 (40.0)	9 (45.0)	10 (50.0)	
Age (year), median (min-max)	21.5 (18.0-27.0)	22.0 (18.0-30.0)	22.0 (18.0-29.0)	0.871**
BMI, (kg/m^2^), median (min-max)	22.4 (17.5-33.8)	24.2 (18.6-27.6)	22.3 (17.3-26.2)	0.664***
ASA, n (%)				0.307*
1	18 (90.0)	14 (70.0)	18 (90.0)	
2	2 (10.0)	6 (30.0)	2 (10.0)	
Time for Aldrete recovery score to reach 9 (minutes), median (min-max)	8.0 (5.0-14.0)	8.0 (4.0-14.0)	5.0 (3.0-10.0)	0.004***
Pain severity just after emerging from anesthesia, n (%)				<0.001*
None	0 (0.0)	4 (20.0)	11 (55.0)	
A little	8 (40.0)	6 (30.0)	4 (20.0)	
Moderate	12 (60.0)	9 (45.0)	5 (25.0)	
Severe	0 (0.0)	1 (5.0)	0 (0.0)	
Complete	0 (0.0)	0 (0.0)	0 (0.0)	
Number of opioid medications needs in the 24 hours postoperatively, median (min-max)	3.0 (2.0-5.0)	3.0 (1.0-4.0)	2.0 (0.0-4.0)	0.004**
Number of rescue analgesic drug medication needs in the 24 hours postoperatively, median (min-max)	0.0 (0.0-1.0)	0.0 (0.0-0.0)	0.0 (0.0-0.0)	<0.001**
Number of patients with and without complications postoperatively (nausea, vomiting, arrhythmia, agitation), n (%)				0.235*
With complication	4 (20.0)	6 (30.0)	3 (15.0)	
Without complication	16 (80.0)	14 (70.0)	17 (85.0)	
Duration of surgery (minute), median (min-max)	285 (210-350)	270 (200-365)	300 (200-360)	0.437**
Maxilla-mandibular movement amount				
Maxillary advancement	4.5 (2.0-8.0)	4.0 (2.0-5.0)	4.0 (2.0-7.0)	0.081**
Mandibular setback	3.5 (0.0-6.0)	4.0 (2.0-6.0)	3.0 (2.0-5.0)	0.238**
Impaction amount	2.0 (0.0-4.0)	2.0 (0.0-4.0)	2.0 (0.0-4.0)	0.623**

VAS values at 30 minutes, 60 minutes, and 24 hours were lower in the paracetamol-tenoxicam group than in the other groups (p<0.001, Table 2).

**Table 2:** Comparison of the postoperative VAS values of the groups according to the measurement times *Kruskal-Wallis analysis test IQR, interquartile range; VAS, visual analogue scale Note: The "a" and "b" superscripts are used to indicate differences between the groups. Groups with the same superscripts are statistically similar.

Measurements	Paracetamol, median (IQR)	Tenoxicam, median (IQR)	Paracetamol+tenoxicam, median (IQR)	p-Value
30 minutes	50.0 (40.0)^a^	40.0 (30.0)^a^	20.0 (20.5)^b^	<0.001*
60 minutes	40.0 (20.0)^a^	30.0 (30.0)^a^	20.0 (20.0)^b^	<0.001*
20 minutes	40.0 (45.0)	50.0 (40.0)	40.0 (40.0)	0.134*
24 hours	20.0 (20.0)^a^	15.0 (10.0)^a^	0.0 (5.0)^b^	<0.001*

In terms of recovery, as indicated by the time taken for the Aldrete recovery score to reach 9, significant differences were observed among the groups (p=0.004). Specifically, the paracetamol-tenoxicam combination group demonstrated a shorter recovery time, with a median of 5.0 minutes (range: 3.0-10.0), compared to both the paracetamol group (median: 8.0 minutes, range: 5.0-14.0) and the tenoxicam group (median: 8.0 minutes, range: 4.0-14.0, Table 1). The need for a rescue analgesic drug in the first 24 hours was not observed in the tenoxicam and paracetamol-tenoxicam groups. Postoperative complications and duration of surgery and performed amounts of maxilla-mandibular movements showed a statistically similar distribution in the groups (p>0.05, Table 1). Vital signs measured at 5, 10, and 15 minutes during the administration of analgesics demonstrated no significant differences among the groups (p>0.05, Table 3).

**Table 3:** Vital signs during analgesic administrations *One-way analysis of variance. **Kruskal Wallis test.

Measurements	Paracetamol, mean (SD)	Tenoxicam, mean (SD)	Paracetamol+tenoxicam, mean (SD)	p-Value
Heart rate				
Pre-administration	82.15 (10.79)	79.65 (10.51)	87.35 (13.9)	0.120*
At 5 minutes	82.9 (12.86)	81.15 (10.03)	89.05 (14.78)	0.128*
At 10 minutes	84.85 (15.1)	84.25 (10.57)	89 (12.37)	0.448*
At 15 minutes	85.3 (14.67)	88.3 (9.24)	88.65 (13.68)	0.706*
Blood pressure				
Pre-administration	71.2 (7.98)	72.6 (11.26)	72.7 (10.93)	0.996**
At 5 minutes	72.6 (7.66)	74.15 (14.45)	71.45 (9.17)	0.785**
At 10 minutes	72.8 (7.77)	77.05 (15.72)	71.55 (9.08)	0.510**
At 15 minutes	73.7 (6.99)	77.15 (12.1)	71.65 (10.04)	0.312*

Confounding factors' influence on the outcomes of the study, which include the VAS scores postoperatively and the number of postoperative opioid consumption, was assessed. According to the analyses, most factors, such as maxillary advancement, maxillary impaction, and mandibular setback, showed no significant correlation with these outcomes (all p>0.05, Table 4). Notably, the duration of surgery was found to have a significant correlation with the VAS score at 30 minutes postoperatively (p=0.048, Table 4).

**Table 4:** Comparison of outcomes with covariates *Point biserial correlation analysis. **Spearman's rho correlation coefficient. BMI, body mass index; ASA, American Society of Anesthesiologists; VAS, visual analogue scale

		VAS score at 30 minutes postoperatively	VAS score at 60 minutes postoperatively	VAS score at 120 minutes postoperatively	VAS score at 24 hours postoperatively	Number of postoperative opioid consumption
Gender	R	0.068	0.012	0.043	0.234	0.077
	p	0.604*	0.927*	0.742*	0.072*	0.558*
ASA	r	-0.076	0.089	-0.043	0.200	0.008
	p	0.564*	0.501*	0.742*	0.126*	0.952*
Age	r	0.099	-0.189	0.106	0.258**	-0.046
	p	0.453**	0.148**	0.420**	0.061**	0.728**
BMI	r	0.104	0.098	0.002	0.063	-0.093
	p	0.431**	0.456**	0.986**	0.630**	0.479**
Maxillary advancement	r	0.072	-0.081	0.009	-0.056	0.017
	p	0.584**	0.536**	0.943**	0.673**	0.896**
Maxillary impaction	r	-0.189	0.079	0.038	-0.101	-0.132
	p	0.147**	0.548**	0.772**	0.442**	0.314**
Mandibular setback	r	0.06	0.169	0.137	0.055	-0.020
	p	0.647**	0.197**	0.298**	0.678**	0.880**
Duration of surgery	r	0.257	0.222	0.217	0.361	0.208
	p	0.048**	0.089**	0.095**	0.012**	0.110**

## Discussion

Methods and approaches in treating postoperative pain have been changing continually with a better understanding of the mechanism of pain. Also, with the knowledge that acute pain has a high potential to transform into chronic pain, the importance given to postoperative pain management has increased in recent years [11].

Today, evidence supports that including NSAIDs in the postoperative analgesia protocol is a practical component of a multimodal, opioid-sparing regime to manage acute pain. As such, it is recommended almost in all ERAS (Enhanced Recovery After Surgery) guidelines [12]. ERAS was established in 2010 to improve surgical outcomes, reduce complications, improve patient experience, and reduce hospital stays. Since then, the ERAS society has created a set of consensus guidelines for various surgeries. Recently, ERAS protocols have been created following the consensus reached after studies specific to each surgical field such as vascular, bariatric, orthopedic, colorectal, gynecological, and many others [13-17]. However, the ERAS protocol for orthognathic surgery is currently unavailable.

There are studies in which NSAIDs are included in the analgesia protocol in double-jaw surgery. As a result of these studies, it was reported that postoperative pain and opioid use were reduced in patients using NSAIDs [18,19]. In addition, Oncul et al. [20] applied paracetamol to one group and NSAID to another group and concluded that both drugs were effective in postoperative pain in double-jaw surgery patients. In the present study, it was observed that postoperative pain decreased significantly in the tenoxicam-paracetamol groups than in the other groups.

Paracetamol and NSAIDs have different mechanisms of action. A systematic review of randomized trials found that the combination of paracetamol with NSAIDs was more effective for postoperative pain after various surgical procedures than paracetamol or NSAIDs alone in 85% and 64% of studies, respectively [7]. In the literature, many studies were found in which NSAIDs and paracetamol were used together after oral surgeries. It was noted that most of these studies included diclofenac sodium or ibuprofen as NSAIDs. Except for the study by Matthews et al., the conclusion of almost all of these studies was that the combinations provide better analgesia than either drug alone [21,22-27]. Considering the postoperative VAS scores and the amount of analgesic drug consumption, it could be concluded that better analgesia was obtained in the combination group in the present study.

The application of an arch-bar and the presence of edema in the soft tissue, which is the area that also includes the respiratory tract, necessitate not performing additional applications, such as opioid applications that will trigger postoperative respiratory depression in double-jaw surgeries. For this reason, we believe that there is a need for an analgesic method to minimize and even reset the need for opioid use, which may cause sedation and respiratory depression in the postoperative period, especially in double-jaw surgeries.

Tenoxicam belongs to the oxicam subgroup of the nonspecific cyclo-oxygenase (COX) inhibitor family. Analgesic and anti-inflammatory activity mainly occurs through the inhibition of prostaglandins in the COX-2 enzyme pathway. Prostaglandins in the COX-1 enzyme pathway are protective prostaglandins for the gastrointestinal and cardiovascular systems. Tenoxicam is 1.34 times more effective in inhibiting COX-2 compared to COX-1 [28]. This means it is both a potent analgesic and anti-inflammatory and has lower gastrointestinal and cardiovascular side effects than other COX-2 inhibitors. Another advantage is that it has a long half-life. Unfortunately, our literature review has found no study in the tenoxicam and orthognathic surgery field.

Limitations

One limitation of our study is the absence of VAS scores collection between 120 minutes and 24 hours. However, this decision was based on specific rationales. The pharmacokinetics and dynamics of the analgesic drugs used in our study typically exhibit their peak effects within the initial hours post-administration. Therefore, by assessing the VAS scores at 30 minutes, first hour, and second hour, we aimed to capture the immediate postoperative analgesic effects provided by the drugs. This approach helped us understand the effectiveness of our pain management strategies in the early postoperative period. Another limitation of this study was that the time of the first analgesic drug administration needed to be recorded. However, according to the results of our research, it was observed that no additional analgesic drug was used according to the VAS scores evaluated up to the postoperative 2 hours of the paracetamol-tenoxicam group.

## Conclusions

In conclusion, the current study revealed that both tenoxicam and the combination of tenoxicam and paracetamol effectively reduce postoperative pain intensity and decrease opioid consumption in patients undergoing double-jaw surgery. However, the paracetamol-tenoxicam combination exhibited certain advantages over tenoxicam alone. Notably, the combined group had a higher proportion of patients experiencing no pain upon emergence from anesthesia, boasted shorter recovery times, and recorded lower VAS scores at 30 minutes, 60 minutes, and 24 hours postoperatively. These outcomes underscore the potential superiority of combining tenoxicam and paracetamol for postoperative analgesia in this patient group.
